# Effect of heat stress on ileal epithelial barrier integrity in broilers divergently selected for high- and low-water efficiency

**DOI:** 10.3389/fphys.2025.1558201

**Published:** 2025-04-07

**Authors:** Elizabeth S. Greene, Brooklee Roach, Maria Fernandez Cuadrado, Sara Orlowski, Sami Dridi

**Affiliations:** ^1^ Division of Agriculture, Center of Excellence for Poultry Science, University of Arkansas, Fayetteville, AR, United States; ^2^ Har-Ber High School, Springdale, AR, United States

**Keywords:** broiler, heat stress, water efficiency, gut integrity, gene expression

## Abstract

Water scarcity and rising global temperatures are two of the greatest current and future threats to poultry sustainability. Therefore, selection for water efficiency (WE) and heat resilience are of vital importance. Additionally, intestinal integrity is of critical importance under challenging conditions to maintain nutrient absorption and therefore, growth and performance of broilers. Here, we examined the effect of chronic cyclic heat stress (HS) on the ileal expression profile of tight-junction, gap-junction, adherens, and desmosome genes in the fourth generation of divergently selected low (LWE)- and high water efficient (HWE)-chicken lines. LWE birds exhibited higher levels of gut permeability, regardless of temperature, as measured by fluorescein isothiocyanate–dextran (FITC-D). Among the claudins (CLDN), *Cldn1* showed greater expression in the HWE as compared to LWE, regardless of temperature. *Cldn5*, -*16*, -*20*, and -*34* genes were all greater in LWE and lower in HWE during HS. Conversely, *Cldn25* was decreased in LWE but increased HWE under HS. *Cldn4* was increased in the HWE line and decreased by HS. Cingulin (*Cgn*) gene expression was lower in HWE as compared to LWE and lower in HS as compared to thermoneutral (TN) condition. Gap junction protein α1 (*Gja1*) and desmoglein 4 (*Dsg4*) were greater in the HWE as compared to the LWE. Cadherin 1 (*Cdh1*) gene expression was greatest in the HWE in TN conditions and lowest in HWE under HS, whereas catenin α2 (*Ctnna2*) and desmocollin 1 (*Dsc1*) were highest in HWE during HS compared to all other groups. This differential expression of key genes associated with intestinal barrier integrity likely contributes to the water efficiency phenotype and the response of these birds to HS.

## 1 Introduction

Water insecurity is a significant current concern worldwide, with the threat of scarcity only projected to rise with global warming. As of 2023, approximately half of the global population faces some form of water insecurity for at least 1 month a year, and one-fourth face extremely high levels of water stress, withdrawing over 80% of their renewable annual freshwater supply (Kuzma et al., 2023). As global temperatures are predicted to rise, and rainfall patterns shift, an ever-increasing segment of the world population will experience water stress (Greve et al., 2018). Combating this rising concern will require action at every level; however, agricultural industries must balance any changes in water usage with potential impacts on productivity. To this end, our research group has generated two lines of chickens divergently selected for water efficiency (ratio of water consumed to body weight gain). Under thermoneutral conditions, these high water efficient (HWE) birds had 3- and 47-points better feed (FCR) and water conversion ratio (WCR), respectively, compared to their low water efficient (LWE) counterparts (Aloui et al., 2024). Under chronic heat stress conditions, the HWE line drank less water and also had better FCR and WCR compared to the LWE birds (1.47 vs. 1.51 for FCR and 2.65 vs. 3.12 for WCR), indicating that they are resilient to heat stress (Aloui et al., 2024).

It has been well established that modern broilers are particularly susceptible to elevated temperatures, with decreases seen in growth, productivity, and livability (Lara and Rostagno, 2013). Virtually all dietary nutrients, including water, are absorbed across the epithelium of the intestine, and the importance of gut health and integrity has become increasingly understood across all species. Heat stress has been shown to negatively impact intestinal morphology (crypt depth, mucous area, and villus height), and leads to decreases in nutrient absorption (Marchini et al., 2011). Breakdown of intestinal integrity, known as leaky gut syndrome, also allows for increased translocation of harmful substances (bacteria, toxins, *etc.*), which is further associated with increased incidence of disease. Intestinal barrier integrity is maintained through the stability of distinct intercellular junctional complexes known as tight junctions, adherens junctions, gap junctions, and desmosomes (Vancamelbeke and Vermeire, 2017). Altered expression or changes in the structure of these complexes result in decreased nutrient absorption, as well as increased passage of ions and water to the intestinal lumen (Barmeyer et al., 2015). However, how these factors may be impacted by selection for water efficiency in poultry has yet to be determined. Therefore, here, we examine the effects of HS on ileal barrier integrity in two lines of broilers divergently selected for high and low water efficiency.

## 2 Materials and methods

### 2.1 Care and use of animals

All animal care and use were conducted in accordance with the recommendations in the guide for the care and use of laboratory animals from the National Institutes of Health. The protocol was approved by the University of Arkansas Animal Care and Use Committee (protocol # 23015). Full details of divergent selection for the LWE and HWE lines and the experimental design have been previously reported (Aloui et al., 2024). Briefly, for the genetic selection program, the base population utilized was a 2015 Modern Random Bred (MRB) population (broiler (meat-type) chickens) and the selection trait was water conversion ratio [WCR = water intake (g)/body weight gain (g)] as described previously (Aloui et al., 2024). On day of hatch, male chicks (240 chicks/line) were individually wing‐banded for line identification and weighed, then placed in 12 controlled environmental chambers in the Poultry Environmental Research Laboratory at the University of Arkansas (2 floor pens/chamber, 6 chambers/line, 20 birds/pen). Each pen was covered with clean pine wood shavings and equipped with separate hanging feeders and a nipple water line attached to a low‐flow water monitoring system (Hiltz et al., 2021). Water and standard diets were provided *ad libitum,* with industry standard rearing temperatures and light cycles. On d29, birds were exposed to thermoneutral (25°C) or chronic cyclic heat stress (36°C for 9 h/day from 9:00 a.m. to 6:00 p.m.) conditions (3 chambers-6 pens-120 birds/line/environment). On d49, birds (n = 12/group) were humanely euthanized by cervical dislocation and approximately 3–4 cm ileum section (∼3 cm anterior to the ileocecal junction) was dissected, snap frozen in liquid nitrogen, and stored at −80°C for further analysis.

### 2.2 Determination of serum fluorescein isothiocyanate-dextran (FITC-D) levels

Serum FITC-D concentrations were determined as previously described (Ruff et al., 2020; Tabler et al., 2020). Briefly, 12 birds from each group were orally gavaged with FITC-D (8.32 mg/kg, MW 3–5 kDa, Sigma-Aldrich, St. Louis, MO). One hour post gavage, blood was collected from the brachial wing vein, serum separated, and fluorescence was measured (Ex 428 nm/Em 528 nm) using the Synergy HTX multi-mode micro plate reader (BioTek, Winooski, VT).

### 2.3 RNA isolation, reverse transcription, and quantitative real-time PCR

Total RNA extraction and RT-qPCR conditions were previously described (Aloui et al., 2024; Tabler et al., 2020; Greene et al., 2024; Dridi et al., 2022). Briefly, total RNA was extracted from the ileum using Trizol reagent (ThermoFisher Scientific, Waltham, MA) according to manufacturer’s protocol. RNA concentration was determined using the Take 3 micro volume plate and Synergy HTX multi-mode microplate reader (BioTek, Winooski, VT). RNAs (1 µg) were reverse transcribed *via* qScript cDNA SuperMix (Quanta Biosciences, Gaithersburg, MD) in a 20-µL total reaction. Real-time quantitative PCR (Applied Biosystems 7,500 Real-Time PCR system) was performed using 5 µL of 10×-diluted cDNA, 0.5 µM of each forward and reverse specific primers for each gene, and SYBR Green Master Mix (ThermoFisher Scientific, Rockford, IL) in a total 20-µL reaction. Oligonucleotide primers specific for chicken were used as previously reported (Tabler et al., 2020), or as provided in Table 1. Relative expression of target genes were determined by the 2^−ΔΔCT^ method (Schmittgen and Livak, 2008), with LWE under TN conditions used as calibrator.

**TABLE 1:** Oligonucleotide qPCR primers.

Gene^a^	Accession number^b^	Primer sequence (5’-3’)	Orientation	Product size, bp
*Cldn1*	NM_001013611.2	CCCACGTTTTCCCCTGAAA	For	61
		GCCAGCCTCACCAGTGTTG	Rev	
*Cldn2*	NM_001277622.1	CCCAGCTGATGGCAAAGG	For	61
		AGGCTGATGGCACCAAAATAGT	Rev	
*Cldn4*	XM_003642382.6	CGAGGTGAGATCCCCGAAA	For	71
		GGGCGTTTGGTGCTCTTCT	Rev	
*Cldn5*	NM_204201.2	ACGTCGTTTTGTTCCGTTGTT	For	57
		CTCAAAGGCGCACAGATCAG	Rev	
*Cldn8*	XM_004938379.5	CCGTGCCAAGTGTTACCAAA	For	148
		CCCTAGGTTTAAATGGGAAGATTTT	Rev	
*Cldn9*	XM_004946417.5	AGCATCGTCACCAACTTCTACAAC	For	64
		CAGCCCCCAGCTCTCTCTT	Rev	
*Cldn10*	NM_001277767.2	CCGCTGTCTGTCTGGGTTTC	For	59
		TGTGCACTTCATCCCAACCA	Rev	
*Cldn11*	XM_040679570.2	TTCCCCCGGTCATCAGTATG	For	62
		GTTACGTATCGCAGCGTTAGGA	Rev	
*Cldn12*	XM_040665727.2	GAGCCTGCCTTCTCCCTTCT	For	67
		AGAGGCATAGCTGTGCATGCT	Rev	
*Cldn14*	XM_015300231.4	GCGGTCTCTGGAGGGATTG	For	58
		AAACGGGTACCAGGCATGTG	Rev	
*Cldn15*	XM_046898719.1	TGGCAGCCTTCACCACCTA	For	63
		CGTGATTCCTTCCACTGCTTCT	Rev	
*Cldn16*	XM_426702.6	GCTCTGGCTTGTTGTAGGTTACAG	For	72
		TGTAGAGCATGAAATCACCTTAGCA	Rev	
*Cldn19*	XM_003642541.5	CACCAAGAGCCGCATTGC	For	57
		CACAGACCGCAGAGGATGAA	Rev	
*Cldn20*	XM_040667274.2	CTCGCAGGAATTTTTGGATTAGTAC	For	71
		TGGTCCAGAAAATTGGAAATGA	Rev	
*Cldn22*	XM_040699650.1	TTCGGTCCATTACGCAGTAACA	For	66
		GGCCTAGTTTCAGTTTCCAAGTG	Rev	
*Cldn23*	XM_004941160.5	TGGGATGTGCTGGAAGATGA	For	87
		GTCACCGTCCTGGAGCTACAG	Rev	
*Cldn25*	XM_004948061.5	CCACCACTCACACCCCAAA	For	58
		CAGCCGAAATCCGCAGTCT	Rev	
Cldn34	XM_040659461.2	GTGGGTGGCTGCTTCTACGT	For	64
		AGAAGTTATGGCTCACTGGAATCAG	Rev	
*Nectin1*	XM_040690205.2	CCGGCAACCGGGAAA	For	65
		GCCCTCCATCCGATTCGT	Rev	
Afdn	XM_040669052.2	TCCGGAAGGACATAGAATACATTG	For	80
		AGATGTGCTAGAATCCACAGATGAAT	Rev	
*Gja3*	NM_001040644.2	GTGGGAAGGCCTGGGTTT	For	76
		TTGCTATTTTCCCCCACTACAAC	Rev	
*Gjb1*	NM_204371.3	ACAAGCAGAACGAGATCAACCA	For	69
		TGCGGCGCAGCATGT	Rev	
*Gjc2*	NM_001199581.2	TGGAGCCCTTAGGATGTTGTG	For	63
		GAGTCGTCGGTGCCTTGGT	Rev	
*Gjd2*	NM_204582.2	GCTGACCGTGGTGGTGATC	For	62
		CGTACACCGTCTCCCCTACAA	Rev	
*Cgn*	NM_001347391.2	CCCTCTTCTTCATGGCTTTTTG	For	131
		CCGAGGGACACAATTGCATA	Rev	
*Cdh2*	NM_001001615.2	GACCCTACAGCCCCACCATA	For	61
		TGGAGCCGCTTCCTTCATAG	Rev	
*Ctnna2*	NM_205136.2	GAATTGCCTCTTCAGAGTTTGCA	For	57
		TCGTGCCCCGCTTCAC	Rev	
*Ctnnb1*	NM_205081.3	TGCCCCACTGCGTGAAC	For	58
		TGCTCTAACCAGCAGCTGAACT	Rev	
*Dsg2*	XM_040664387.2	AAGCTGTCTCTTTTGCGGAAGA	For	73
		TCCCCCTGAGAATAAACCAGAA	Rev	
*Dsg4*	XM_040664432.2	CGTGCAATACTCCAGCCAGTAA	For	73
		GCTAATAAGTGTTGGTGCAAGTTTCA	Rev	
*Dsc1*	XM_040664420.2	TGGATTATGAAAATGCCAAACAA	For	67
		AGCATGTAGGGTGCCTCATTG	Rev	

^a^ AFDN, afadin; CDH2, cadherin 2; CGN, cingulin; CLDN, claudin; CTNNA2, Catenin α2; CTNNB1, Catenin β1; DSC1, desmocollin 1; DSG2, desmoglein 2; DSG4, desmoglein 4; GJA3, gap junction protein α3; GJB1, gap junction protein β1; GJC2, gap junction protein γ2; GJD2, gap junction protein δ1.

^b^ Accession number refers to GenBank (National Center for Biotechnology Information–NCBI).

### 2.4 Western blot

Western blot was performed as previously described (Lassiter et al., 2015). Briefly, ileal tissue was homogenized in lysis buffer containing protease- and phosphatase-inhibitors. Protein concentrations were determined *via* Bradford assay (Bio-Rad, Hercules, CA) and the Synergy HTX multimode microplate reader (BioTek, Winooski, VT). Proteins were separated on 4%–12% Bis-Tris gels (Life Technologies, Carlsbad, CA), and transferred to PVDF membranes. Membranes were blocked with 5% non-fat milk in TBS-T for 1 h at room temperature, then incubated with primary antibodies overnight at 4°C. Primary antibodies used were rabbit anti-claudin 4 (CLDN4, 1:1,000, bs-2790R, Bioss, Woburn, MA), rabbit anti-CLDN5 (1:1,000, sc-28670, Santa Cruz Biotechnology, Dallas, TX), and anti-rabbit zona occluding-2 (ZO-2, 1:1,000, 38–9,100, ThermoFisher Scientific, Waltham, MA). Rabbit anti-Glyceraldehyde 3-phosphate dehydrogenase (GAPDH, 1:1,000, NB300-327, Novus Biologicals, Centennial, CO) was used as a loading control. Horseradish peroxidase (HRP)-conjugated secondary antibody (goat anti-rabbit IgG #7074, Cell Signaling, Danvers, MA) was used at 1:5,000 dilution for 1 h at room temperature. The signal was visualized by chemiluminescence (SuperSignal West Femto Maximum Sensitivity Substrate, ThermoFisher Scientific, Waltham, MA) and captured by FluorChem M MultiFluor System (ProteinSimple, Santa Clara, CA). Image acquisition and analysis were performed with AlphaView software (version 3.4.0.0, ProteinSimple, Santa Clara, CA).

### 2.5 Statistics

Gene and protein expression data (n = 12/line/environment) were analyzed by two‐way ANOVA. When ANOVA revealed significant interaction effects, the means were compared by Tukey’s HSD multiple comparison test. If the line by environment interaction was not significant, the main effect (Line, L or Environment, E) was analyzed separately by Student’s *t*‐test test using Graph Pad Prism version 9.00 for Windows (Graph Pad Software, La Jolla California, USA). Data are presented as the mean ± standard error of the mean and the statistical significance was set at *P* < 0.05.

## 3 Results

### 3.1 Intestinal permeability is decreased in HWE as compared to LWE broilers

**FIGURE 1:**
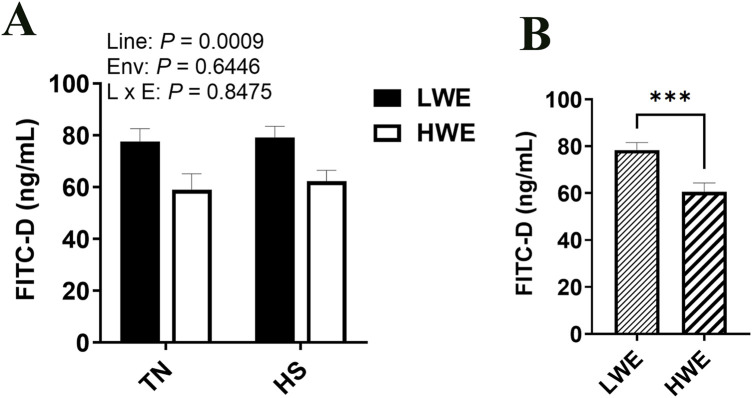
Effect of heat stress on serum FITC-D levels in LWE and HWE chickens. **(A, B)** Data were analyzed by two-way ANOVA and are presented as mean ± SEM (n = 12 birds/group). When the line by environment interaction was not significant, the main effect was analyzed separately by Student’s *t*‐test. *** indicates significant difference at *P* < 0.001. E and Env, environment; HS, heat stress; HWE, high water efficient; L, line; LWE, low water efficient; TN, thermoneutral.

As measured by FITC-D in serum, intestinal permeability was lower in the HWE as compared to the LWE birds (*P* = 0.0009, Figures 1A, B). There was no overall effect of HS (Figure 1A).

### 3.2 Differential expression of tight junction proteins in heat-stressed LWE and HWE broilers

**FIGURE 2:**
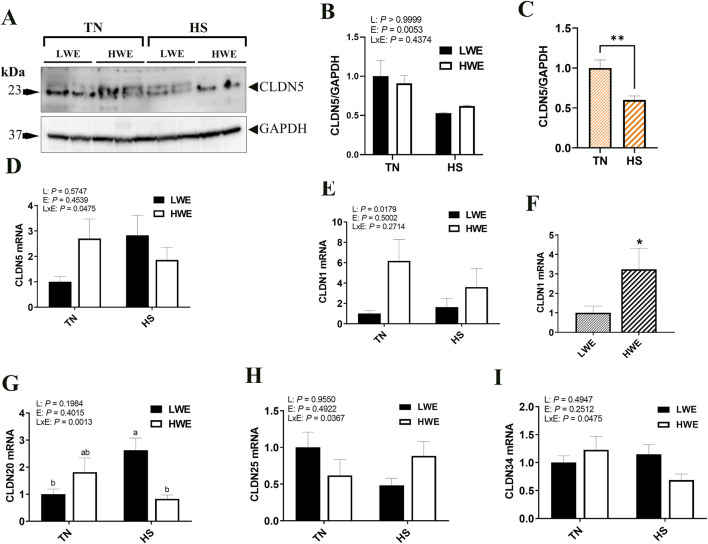
Effect of heat stress on the expression of barrier-forming claudins in LWE and HWE chickens. Protein expression of CLDN5 was determined by Western blot. **(A–C)** Gene expression of CLDN5 **(D)**, CLDN1 **(E, F)**, CLDN20 **(G)**, CLDN25 **(H)**, and CLDN34 **(I)** were measured by qPCR. Data were analyzed by two-way ANOVA and are presented as mean ± SEM (n = 12 birds/group). When the line by environment interaction was not significant, the main effect was analyzed separately by Student’s *t*‐test. Different letters indicate significant differences at *P* < 0.05. * and ** indicate significant difference at *P* < 0.05 and *P* < 0.01, respectively. CLDN, claudin; HS, heat stress; HWE, high water efficient; LWE, low water efficient; TN, thermoneutral.

For the barrier-forming claudins, CLDN5 protein levels were significantly decreased by HS compared to TN conditions (Figures 2A–C). The expression of *Cldn5* gene, however, was upregulated in LWE, and downregulated in HWE under HS conditions, which resulted in a significant line by environment interaction (Figure 2D). The expression of *Cldn1* gene was significantly upregulated in the ileum of HWE birds compared to their LWE counterparts (Figures 2E, F). *Cldn20* mRNA abundances were induced by HS only in LWE but not in HWE birds, resulting in a significant line by environment interaction (Figure 2G). Heat stress downregulated *Cldn25* gene expression in LWE and *Cldn34* in HWE birds, causing a significant line by environment interactions (*P* = 0.0367 and *P* = 0.0475 for *Cldn25* and *Cldn34*, respectively, Figures 2H, I). There was no significant effect of line or of HS on the ileal expression of *Cldn8*, *Cldn9*, and *Cldn22* (Table 2).

**TABLE 2:** Relative expression of ileal integrity-associated genes in heat-stressed LWE and HWE broilers.

Environment^a^	TN		HS		*P* value		
Gene^b^/Line^c^	LWE	HWE	LWE	HWE	L	E	L x E
Barrier-forming CLDNs							
*Cldn8*	1 ± 0.20	2.24 ± 0.75	2.16 ± 0.63	2.01 ± 0.82	0.4476	0.5205	0.3395
*Cldn9*	1 ± 0.17	1.38 ± 0.41	1.62 ± 0.58	1.30 ±0.38	0.9475	0.5294	0.4181
*Cldn22*	1 ± 0.14	1.91 ± 0.68	1.47 ± 0.24	1.85 ± 0.42	0.1429	0.6330	0.5357
Pore-forming CLDNs							
*Cldn2*	1 ± 0.33	0.81 ± 0.32	0.75 ± 0.27	0.73 ± 0.20	0.7169	0.5799	0.7786
*Cldn19*	1 ± 0.24	1.09 ± 0.27	1.31 ± 0.33	0.79 ± 0.22	0.4528	0.9777	0.2713
*Cldn23*	1 ± 0.09	1.02 ± 0.11	0.74 ± 0.05	0.94 ± 0.07	0.2270	0.0682	0.3557
Other TJ proteins							
*ZO-3*	1 ± 0.32	5.11 ± 2.20	1.81 ± 0.76	2.53 ± 1.11	0.6883	0.1208	0.4323
*Ocln*	1 ± 0.29	1.06 ± 0.27	0.91 ± 0.27	0.48 ± 0.06	0.4613	0.1812	0.3171
*Patj*	1 ± 0.44	2.17 ± 0.72	1.14 ± 0.27	1.95 ± 1.28	0.2671	0.9697	0.8402
*Jama*	1 ± 0.37	2.21 ± 0.84	1.35 ± 0.82	0.50 ± 0.14	0.7788	0.2938	0.1123
Gap junctions							
*Gja3*	1 ± 0.14	1.65 ± 0.57	1.71 ± 0.22	2.15 ± 0.56	0.2009	0.1627	0.8032
*Gjb1*	1 ± 0.14	0.93 ± 0.24	0.91 ± 0.12	1.18 ± 0.25	0.6212	0.6882	0.4334
*Gjc1*	1 ± 0.30	3.73 ± 1.25	2.62 ± 0.92	2.52 ± 0.77	0.1577	0.8193	0.1306
*Gjc2*	1 ± 0.11	1.21 ± 0.31	1.10 ± 0.23	0.69 ± 0.16	0.6540	0.3557	0.1659
*Gjd2*	1 ± 0.19	1.22 ± 0.40	1.13 ± 0.23	1.98 ± 0.58	0.1823	0.2667	0.4414
Adherens							
*Cdh2*	1 ± 0.17	1.36 ± 0.27	1.01 ± 0.18	1.12 ± 0.18	0.2779	0.6007	0.5400
*Ctnnb1*	1 ± 0.09	0.81 ± 0.07	1.01 ± 0.08	1.01 ± 0.07	0.2634	0.2071	0.2990
*Nectin1*	1 ± 0.08	0.97 ± 0.11	1.16 ± 0.12	0.94 ± 0.07	0.2601	0.5185	0.3590
*Afdn*	1 ± 0.21	0.92 ± 0.16	1.00 ± 0.12	1.24 ± 0.26	0.6852	0.4217	0.4352
Desmosomes							
*Dsg2*	1 ± 0.19	1.42 ± 0.28	1.18 ± 0.12	1.30 ± 0.25	0.4857	0.8842	0.2312

^a^ HS, heat stress; TN, thermoneutral.

^b^ *Afdn*, afadin; *Cdh2*, cadherin 2; *Cldn*, claudin; *Ctnnb1*, beta-catenin; *Dsg2,* desmoglein; *Gja3*, gap junction protein alpha 3; *Gjb1*, gap junction protein beta 1; *Gjc1*, gap junction protein gamma 1; *Gjd2*, gap junction protein delta 2; *Jama*, junctional adhesion molecule A; *ocln*, occludin; *Patj*, PALS1-associated tight junction protein; *Z O -3*, Zonula occludens protein 3.

^c^ HWE, high water efficient; LWE, low water efficient.

**FIGURE 3:**
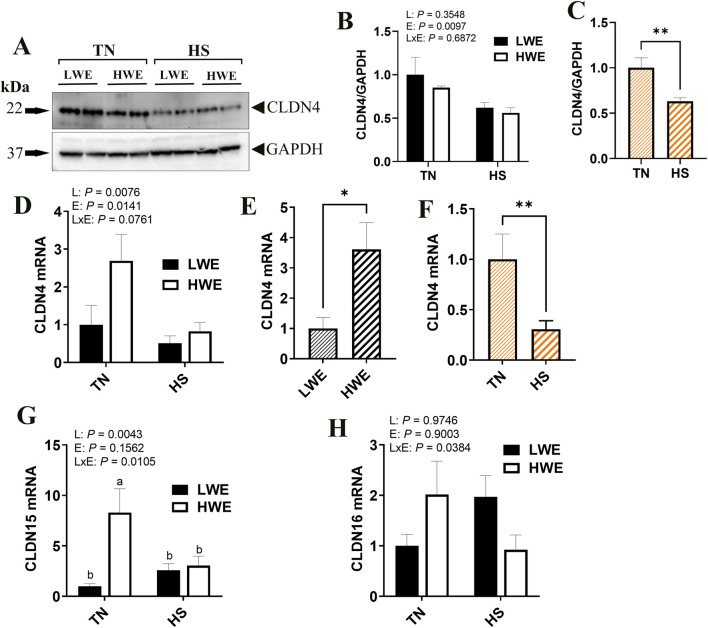
Effect of heat stress on the expression pore-forming claudin expression in LWE and HWE chickens. Protein expression of CLDN4 was determined by Western blot. **(A–C)** Gene expression of CLDN4 **(D–F)** CLDN15 **(G)**, and CLDN16 **(H)** were measured by qPCR. Data were analyzed by two-way ANOVA and are presented as mean ± SEM (n = 12 birds/group). When the line by environment interaction was not significant, the main effect was analyzed separately by Student’s *t*‐test. Different letters indicate significant differences at *P* < 0.05. * and ** indicates significant difference at *P* < 0.05 and *P* < 0.01, respectively. CLDN, claudin; HS, heat stress; HWE, high water efficient; LWE, low water efficient; TN, thermoneutral.

Among the pore-forming claudins, *Cldn4*, *Cldn15*, and *Cldn16* were differentially regulated. *Cldn4* gene expression was affected by both line and environment, where mRNA abundances were increased in the HWE line (*P* = 0.0330) and decreased in HS condition (*P* = 0.0138, Figures 3D–F). CLDN4 protein levels were significantly reduced by HS (Figures 3A–C), although the overall effect of line was not statistically and significantly discerned (*P* = 0.3548). The expression of *Cldn15* gene was higher in the HWE line under TN conditions, and significantly downregulated by HS in the same line, but not in the LWE counterparts (*P* = 0.0043, Figure 3G), resulting in a significant line by environment interaction (*P* = 0.0105). The expression of *Cldn16* gene was upregulated by HS in the LWE and downregulated in the HWE, with a significant line by environment interaction (*P* = 0.0384, Figure 3H). There were no significant effects of line nor environment on *Cldn2*, *Cldn19*, or *Cldn23* (Table 2).

**FIGURE 4:**
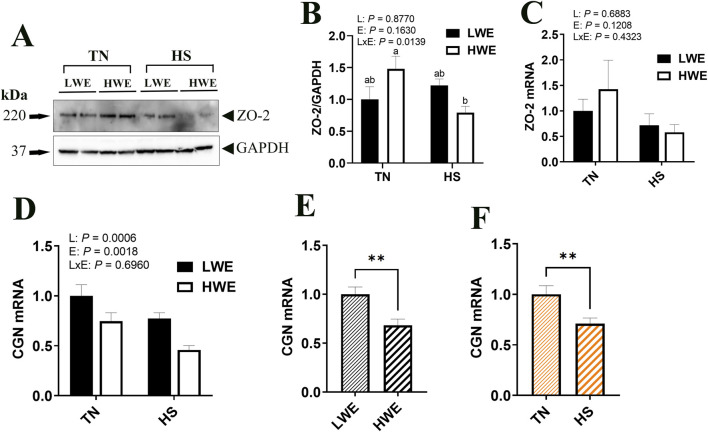
Effect of heat stress on tight-junction gene expression in LWE and HWE chickens. Protein expression of Zo-2 was determined by Western blot **(A, B)**. Gene expression of ZO-2 **(C)** and CGN **(D–F)** was measured by qPCR. Data were analyzed by two-way ANOVA and are presented as mean ± SEM (n = 12 birds/group). When the line by environment interaction was not significant, the main effect was analyzed separately by Student’s *t*‐test. Different letters indicate significant differences at *P* < 0.05. ** indicates significant difference at *P* < 0.01. CGN, cingulin; HS, heat stress; HWE, high water efficient; JAM-A, junctional adhesion molecule A; LWE, low water efficient; OCDN, occludin; PATJ, protein associated to tight junctions; TN, thermoneutral; ZO, zona occludin.

Protein levels of ZO-2 were significantly decreased by HS only in HWE but not in LWE birds, which resulted in a significant line by environment interaction (Figures 4A, B). The gene expression of *Zo-2* remained unchanged (Figure 4C). Cingulin (*Cgn)* gene expression was affected by both line and environment (Figures 4D–F), where it was lower in HWE as compared to LWE (*P* = 0.0006) and lower in HS as compared to TN (*P* = 0.0018). The expressions of Occludin (*Ocln*), zona occludin-3 (*Zo-3)*, PALS1-associated tight junction protein (*Patj*), and junctional adhesion molecule A (*Jam-A*) genes were all unaffected by line or environmental conditions (Table 2).

### 3.3 Differential expression of gap junction gene expression in LWE and HWE broilers during HS

**FIGURE 5:**
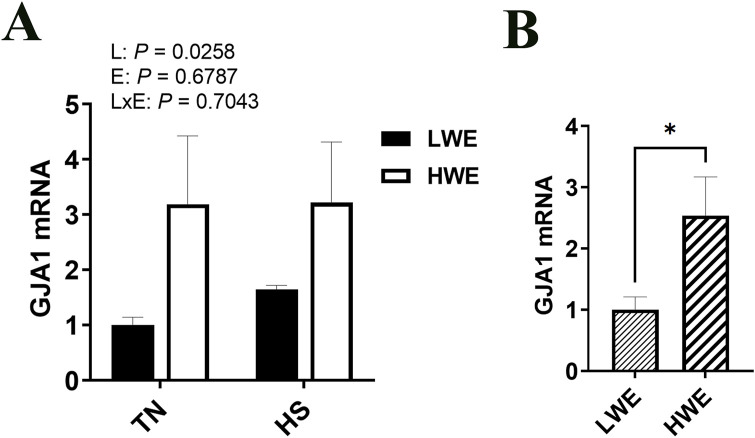
Effect of heat stress on GJA1 gene expression in LWE and HWE chickens. Gene expression of GJA1 **(A, B)** was measured by qPCR. Data were analyzed by two-way ANOVA and are presented as mean ± SEM (n = 12 birds/group). When the line by environment interaction was not significant, the main effect was analyzed separately by Student’s *t*‐test. * indicates significant difference at *P* < 0.05. GJA, gap junction protein α; HS, heat stress; HWE, high water efficient; LWE, low water efficient; TN, thermoneutral.

Abundances of the gap junction protein α1 (*Gja1*) mRNA were affected by line, with a significant increase in the HWE as compared to the LWE birds under both environmental conditions (*P* = 0.0258, Figures 5A, B). There were no effects of line nor environment on gene expression of gap junction protein α3 (*Gja3*), gap junction protein β1 (*Gjb1*), gap junction protein γ1 (*Gjc1*), gap junction protein γ2 (*Gjc2*), or gap junction protein δ1 (*Gjd2*) (Table 2).

### 3.4 Differential expression of adherens junction gene expression in LWE and HWE broilers during HS

**FIGURE 6:**
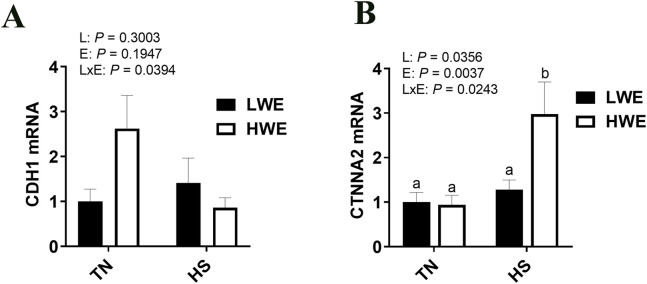
Effect of heat stress on CDH1and CTNNA2 gene expression in LWE and HWE chickens. Gene expression of CDH1 **(A)** and CTNNA2 **(B)** was determined by qPCR. Data were analyzed by two-way ANOVA and are presented as mean ± SEM (n = 12 birds/group). CDH, cadherin; CTNNA2, catenin α2; HS, heat stress; HWE, high water efficient; LWE, low water efficient; TN, thermoneutral.

There was a significant line by environment interaction effect on cadherin 1 (*Cdh1, P =* 0.0394*,* Figure 6A) and Catenin α2 (*Ctnna2*, *P* = 0.0243, Figure 6B) gene expression. Cadherin one expression was higher in HWE birds under TN condition and was downregulated by HS exposure (Figure 6A). Catenin α2 expression was induced by HS only in the ileum of HWE birds (Figure 6B). There were no significant effects of HS nor line on cadherin 2 (*Cdh2*), Catenin β1 (Ctnnb1), Nectin1, or afadin (*Afdn)* (Table 2).

### 3.5 Differential expression of desmosome gene expression in LWE and HWE broilers during HS

**FIGURE 7:**
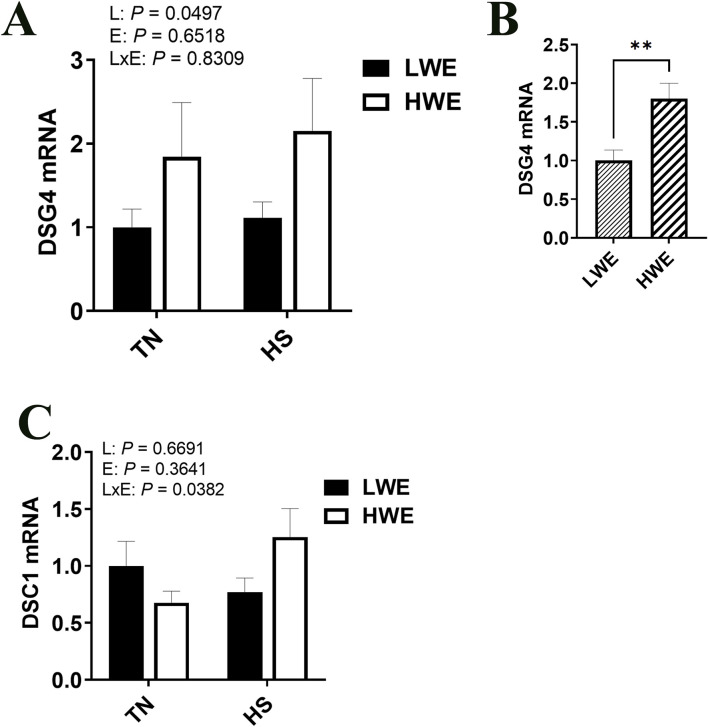
Effect of heat stress on desmosome gene expression in LWE and HWE chickens. Gene expression of DSG4 **(A, B)** and DSC1 **(C)** was measured by qPCR. Data were analyzed by two-way ANOVA and are presented as mean ± SEM (n = 12 birds/group). When the line by environment interaction was not significant, the main effect was analyzed separately by Student’s *t*‐test. ** indicates significant difference at *P* < 0.01. DSC1, desmocollin 1; DSG, desmoglein; HS, heat stress; HWE, high water efficient; LWE, low water efficient; TN, thermoneutral.

Gene expression of desmoglein 4 (*Dsg4*) was increased in the HWE line as compared to the LWE (*P* = 0.0497, Figures 7A, B). There was an interactive effect of line and environment on desmocolin 1 (*Dsc1*), where gene expression was increased by HS only in the HWE line (*P* = 0.0382, Figure 7C). There was no significant effect of HS nor line on desmoglein 2 (*Dsg2*) gene expression (Table 2).

## 4 Discussion

Maintenance of intestinal barrier integrity is critical to organismal health and allows selective permeability of transport of ions, nutrients, and water while restricting pathogens (Rescigno, 2011). Over 80 proteins have been identified as contributing to this barrier, with distinct as well as coordinated functions (Tsukita et al., 2001). A key point, however, is that none of these tight junctions, adherens junction, gap junctions, and desmosome proteins work in isolation. The complex constantly adapts to changes in the biological state through constant remodeling and intracellular trafficking, where proteins are inserted and internalized from the membrane (Shen et al., 2008). Together, they form a well-organized matrix to provide a barrier that is both functional and efficient. Here, regardless of temperature, the HWE birds displayed lower intestinal permeability, as measured by serum FITC-D, compared to the LWE line. As the intestinal barrier integrity is controlled by a complex network of tight junction, gap junction, and desmosomes, here we sought to further assess the expression of barrier integrity-related genes in these two lines of birds under HS conditions.

Claudins are tight junction proteins that can be broadly classified into two categories: barrier-forming and channel- or pore-forming. The barrier-forming claudins restrict the passage of small molecules and electrolytes, whereas the pore-forming claudins allow the passage of ions and water through paracellular channels (Krause et al., 2008). One of the first claudins isolated (Furuse et al., 1998), CLDN1 is highly expressed across the entire intestinal tract, and downregulation of CLDN1 has been shown to be associated with increased intestinal permeability *via* the NFκB pathway (Zhou et al., 2015). Here, the *Cldn1* gene was increased in the ileum of the HWE birds as compared to their LWE counterparts, which likely contributes to the improvements seen in intestinal permeability in this line. Additionally, chronic stress has been associated with decreased intestinal CLDN1 (Zheng et al., 2017). CLDN1 was not significantly affected by HS in the current study, which might indicate an adaptation to the chronic (20 days) HSs. *Cldn5* and *Cldn20* genes showed similar patterns of expression in the two studied lines, with increased expression in the LWE line and decreased expression in the HWE during HS. As barrier-forming claudins, increased expression of these genes tends to decrease membrane permeability (Rahner et al., 2001; Martin et al., 2013), which is supported by the downregulation of *Cldn5* in HS conditions. However, little is known about their function in the avian intestine. CLDN5 is tightly regulated at the blood-brain-barrier and is compromised in several neurological diseases (Hashimoto et al., 2023), while in breast-cancer models, overexpression of CLDN20 increases trans-epithelial electrical resistance (TEER) (Martin et al., 2013), both tissue-specific roles highlighting the complex nature of claudins. Similarly, the function of CLDN25 has yet to be fully understood. A paralog of CLDN22, CLDN25 does not seem to be either directly barrier- or pore-forming; however, its role in barrier regulation seems to depend on the composition of other claudins within the tight junction (Hashimoto et al., 2024). This is supported by evidence of differential effects in different cell types *in vitro*. In mouse brain endothelial cells (bEnd.3), CLDN25 knockout lowered solute permeability, while it increased in Madin-Darby canine kidney strain II (MDCKII) cells. However, TEER was increased in both cell types. Future functional studies are necessary to delineate the function of CLDN25 in the chicken intestine. Likewise, CLDN34 is understudied in both mammalian and avian species. Based on studies in fish, enhanced intestinal expression is related to enhanced immune function, whether in response to a bacterial challenge (Cao et al., 2023) or a dietary immunomodulator (Zhang et al., 2024). Taken together, these data suggest that differential expressions of the barrier-forming claudins are integral components of the differences seen in water efficiency selected broilers; however, further research is necessary to delineate the functions of individual claudins, as well as their interaction in poultry intestine.

Multiple pore-forming claudins are also differentially regulated between the two lines. CLDN2, 10b, 15, 16 and 21 have been shown to form cation-selective paracellular channels, whereas CLDN10a and 17 are understood to form anion-selective channels (Günzel and Yu, 2013). Of particular interest to this work, CLDN2 and 15 are known to move water as well as other solutes such as sodium and potassium (Rosenthal et al., 2020; Samanta et al., 2018). Interestingly, *Cldn2* gene was unaffected in this study; however, its protein expression and localization within the gut are known to be negatively regulated by *Cgn* (Guillemot et al., 2012), a gene that was lower in the HWE line. *Cldn15* showed a greater mRNA expression in the HWE line, particularly under the TN environment. Together, these may be significant contributing factors to the improved water efficiency in this line, as water uptake at the small intestine may be more easily facilitated. Interestingly, CLDN15 also indirectly enhances glucose uptake *via* Na^+^ flux into the lumen of the intestine and subsequently enhances the activity of Na^+^-driven glucose transporter SGLT1 (Tamura et al., 2011), an additional role that may be impacting the BWG and FCR improvements seen in the HWE line (Aloui et al., 2024). CLDN16 is recognized for its role in calcium transport, as mutated CLDN16 may be responsible for defective absorption of Ca^2+^ along the intestine (Weber et al., 2001). Although calcium metabolism has yet to be explored in these lines of birds, it is plausible, based on the differential expression of *Cldn16* due to line × environment interaction*,* observed here, that these lines had different Ca^2+^ transport, absorption, and/or metabolism. Additionally, in chickens, CLDN16 has been localized to goblet cells, suggesting a further role in mucus secretion (Ozden et al., 2010) and thereby homeostasis of intestinal flora. CLDN4 has a unique function, as it is considered pore-forming regulating, as it can interfere with and regulate CLDN2, 7, 15, and 19, and increase the complexity of tight junctions (Van Itallie et al., 2001; Shashikanth et al., 2022a; Shashikanth et al., 2022b), a role which may be indicated in the increased expression in the HWE line. Downregulation of CLDN4 has been seen in areas of intestinal inflammation (Prasad et al., 2005), which is likely reflected here in the lower expression seen in HS, regardless of line. Although the role and regulation of avian ZO-2 are not well known, its ileal downregulation by HS exposure, at least in HWE line here opposed that in the duodenum of broilers from the 1990s (Tabler et al., 2020), which suggests a tissue- or strain-specific regulation (Santos et al., 2021). Based on previous studies showing that rye diet alters gut integrity and induces leaky gut syndrome in broilers (Baxter et al., 2019), and this was accompanied by an upregulation of ileal ZO-2, it is logical to postulate that ZO-2 downregulation in our study supports a better gut integrity in HWE line.

Gap junctions are important in cell structure as well as for cell-to-cell communication *via* the transfer of ions and small molecules between adjacent cells (Wong et al., 2019). Gap junction genes encode connexins, which are differentially and widely expressed throughout the body. Here, among the gap junction genes, only *Gja1* (encoding connexin 43) was differentially expressed, and was higher in the HWE line. GJA1 plays multiple roles in intestinal epithelial health that may be influencing the physiology of the HWE line. First, GJA1 likely impacts intestinal motility *via* interaction with intestinal nerve transmission (Daniel et al., 2001; Daniel and Wang, 1999), as well as its function as a necessary component for motile cilia (Jang et al., 2022). In these broilers, it may be altering motility or gastrointestinal transit time in such a manner that could be influencing increases in water and nutrient absorption and contributing to the water efficiency, BWG, and FCR improvements seen in the HWE line (Aloui et al., 2024). However, digestibility studies have yet to be conducted in these birds, so this remains speculative. Second, connexin 43 is redistributed within intestinal epithelial cells to the basolateral surface during inflammation (as seen in inflammatory bowel disease) (Al-Ghadban et al., 2016), and it has been shown to be associated with inflammasome (de)activation (Roger et al., 2023). The higher expression of *Gja1* here and the downregulation of *Nlrc3* in HWE blood (Greene et al., 2024), suggest that GJA1 might improve the gut inflammatory status of HWE line, resulting in better barrier integrity and better growth performance. In addition, connexins have a short half-life of only a few hours (Segretain and Falk, 2004), so investigating the spatio-temporal nature of their responses to HS in poultry gut is warranted.

Adherens junctions organize and stabilize the condensed actin filaments of the cytoskeleton with the plasma membrane and are assembled from classical cadherins, armadillo proteins, and cytoskeletal adaptor proteins (Harris and Tepass, 2010). E-cadherin (encoded by *Cdh1*) is the most crucial cadherin present on the epithelial surface responsible for adherens junction formation *via trans* adhesive homodimers with other cadherins (Gumbiner, 2005; Brasch et al., 2012). Among the cadherins studied, we saw differential regulation of Cdh1, which was greater in the HWE in TN conditions, but lower under HS. Others have reported increases in *CDH1* in poultry intestine in response to HS; however, the response is likely region-specific, with increases seen in the jejunum, but not in the ileum (Varasteh et al., 2015). Cadherins bind to catenins as part of the stabilization structure, and in particular, α-catenin binds to the E-cadherin/β-catenin complex (Drees et al., 2005). The dimerization of CTNNA2 influences binding to other proteins; monomeric α-catenin binds β-catenin, but not actin, while homodimeric α-catenin binds actin but not β-catenin (Yamada et al., 2005). In this way, it helps regulate microtubule dynamics and has been shown to play a central role in cytoskeletal rearrangement in response to extracellular events (Perez-Moreno and Fuchs, 2006; Arbore et al., 2022). In a fish model, increased methylation on *Ctnna2* was associated with increased intestinal integrity and decreased inflammation (Dhanasiri et al., 2020). Interestingly, *Ctnnna2* expression is also associated with climate adaptation in mediterranean cattle (Flori et al., 2019). As this gene was upregulated only during HS in the HWE line, it may serve a role in helping stabilize the adherens junction, as other components (particularly the cadherins) are unchanged or downregulated as compared to TN conditions.

Desmosomes are the least well studied of the epithelial barrier regulating proteins, but have been shown to create strong intercellular adhesion, particularly in tissues subject to mechanical stress. Intercellular adhesion is normally initiated by adherens junctions, then stabilized by desmosomes *via* connecting intermediate filaments to the plasma membrane (Vasioukhin et al., 2000). Desmogleins form heterodimers with desmocollins (Getsios et al., 2004), and one of the strongest binding has been found between DSC1 and DSG4 (Harrison et al., 2016), both of which show greater expression in the HWE during HS, which is likely contributing to increased intestinal integrity and lower leaky gut. Desmosomes also can exist in two adhesive states, a weaker, Ca^2+^ dependent state, and a stronger, Ca^2+^ independent state termed “hyperadhesion”. This hyperadhesion is critically important in the ability of cells to resist shear forces that would separate cells and disrupt tissues (Garrod and Tabernero, 2014; Beggs et al., 2022). Protein Kinase C (PKC) pathways, which have been shown to be induced by HS (Yang et al., 2007), negatively regulate hyperadhesion (Garrod et al., 2008), a further mechanism by which HS can impact intestinal barrier integrity.

It is interesting to note that both serum FITC-D and many of the measured genes are minimally affected by HS in either line. As these birds were subject to cyclic HS for 20 days, it is plausible that some of the responses are reflective of adaptation to the environmental challenge. It is also plausible that birds were recovered during the cool phase. As the HWE, overall, responded more favorably to HS (in terms of decreased intestinal permeability, and increased growth, water and feed efficiency), it may be that the genes associated with intestinal integrity in the LWE are upregulated to try to fix or improve the disrupted barrier, while the HWE were overall more resilient. It is also possible that although gene and/or protein levels of the tight junctions are unaffected, post-translational modifications (Shigetomi and Ikenouchi, 2017) or distribution/localization within the cell, and therefore function, may be affected by genetics or HS. Indeed, this has been seen in other models, including Llc‐Pk1 kidney cells (Ikari et al., 2005), Caco-2 intestinal epithelial cells (Han et al., 2003), and isolated T cells (Voges et al., 2024), and warrants further investigation in these lines.

Overall, these results provide further evidence for the positive potential of water efficiency selection in poultry, as there were improvements in intestinal integrity over the LWE line, with no additional impairments due to HS. In addition, differential gene expression of intestinal barrier proteins may help delineate some of the underlying mechanisms of improved water efficiency and identify potential markers for further selection.

## Data Availability

The data that support the findings of this study are available from the corresponding author upon reasonable request.
